# Synthesis, Characterization, and Bioactivity of Schiff Bases and Their Cd^2+^, Zn^2+^, Cu^2+^, and Ni^2+^ Complexes Derived from Chloroacetophenone Isomers with S-Benzyldithiocarbazate and the X-Ray Crystal Structure of S-Benzyl-****β****-N-(4-chlorophenyl)methylenedithiocarbazate

**DOI:** 10.1155/2013/362513

**Published:** 2013-11-11

**Authors:** Mohammed Khaled bin Break, M. Ibrahim M. Tahir, Karen A. Crouse, Teng-Jin Khoo

**Affiliations:** ^1^School of Chemical Sciences and Food Technology, Universiti Kebangsaan Malaysia (UKM), Selangor, 43600 Bangi, Malaysia; ^2^Department of Chemistry, Universiti Putra Malaysia, 43400 Selangor, Malaysia; ^3^School of Pharmacy, University of Nottingham, Malaysia Campus, 43500 Selangor, Malaysia

## Abstract

Two bidentate Schiff base ligands having nitrogen sulphur donor sequence were derived from the condensation of S-benzyldithiocarbazate (SBDTC) with 2-chloroacetophenone and 4-chloroacetophenone to give S-benzyl-**β**-N-(2-chlorophenyl)methylenedithiocarbazate (NS2) and S-benzyl-**β**-N-(4-chlorophenyl)methylenedithiocarbazate (NS4) isomers. Each of the ligands was then chelated with Cd^2+^, Zn^2+^, Cu^2+^, and Ni^2+^. The compounds were characterized via IR spectroscopy and melting point while the structure of NS4 was revealed via X-ray crystallography. Finally, the compounds were screened for antimicrobial activity to investigate the effect that is brought by the introduction of the chlorine atom to the benzene ring. X-ray crystallographic analysis showed that the structure of NS4 is planar with a phenyl ring that is nearly perpendicular to the rest of the molecules. The qualitative antimicrobial assay results showed that NS4 and its complexes lacked antifungal activity while Gram-positive bacteria were generally inhibited more strongly than Gram-negative bacteria. Furthermore, NS4 metal complexes were inhibited more strongly than the ligand while the opposite was seen with NS2 ligand and its complexes due to the partial solubility in dimethyl sulfoxide (DMSO). It was concluded that generally NS2 derivatives have higher bioactivity than that of NS4 derivatives and that the Cd complexes of both ligands have pronounced activity specifically on *K. rhizophila*.

## 1. Introduction

The past few decades have seen a growing interest in transition metal complexes of Schiff base ligands as there have been several studies done on complexes that have nitrogen-sulfur donor ligands [1] with particular emphasis on ligands derived from dithiocarbazates, NH_2_NHCS_2_ [2].

SBDTC is interesting due to the fact that its derivatives have the potential to be modified in various ways by introducing several different substituents [1, 3]; moreover, SBDTC-derived Schiff bases have been found to possess anticancer and antimicrobial activities [1, 4].

The extensive literature review that has been made revealed that there were no studies performed on the properties and biological activities of Schiff base ligands and complexes derived from SBDTC, possessing a benzene ring with different halogen isomers. Therefore, our studies were dedicated to form ligands by condensation of 2-chloroacetophenone with SBDTC (NS2) and the condensation of 4-chloroacetophenone with SBDTC (NS4). This was done in order to investigate the changes in properties and biological activities brought about by the position of the chlorine atom on the benzene ring.

**Figure 1:**
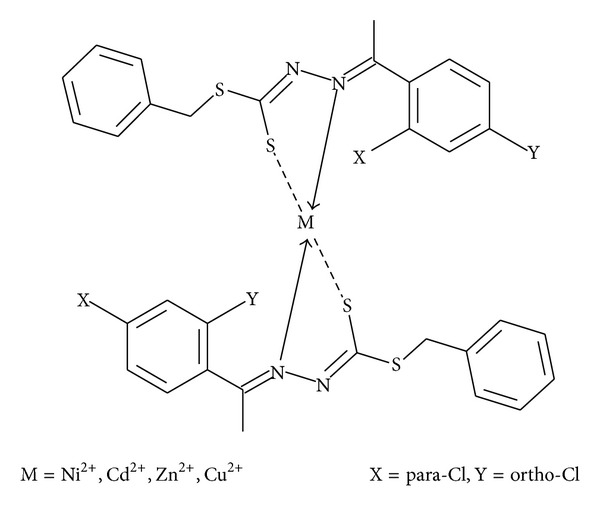
Proposed structure of the complexes.

**Scheme 1:**
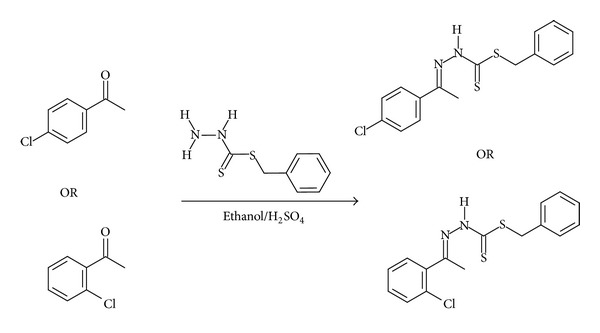
Schematic diagram of the synthesis procedure for NS4 and NS2 ligands.

The synthesized Schiff bases were chelated with Zn^2+^, Cu^2+^, Cd^2+^, and Ni^2+^ salts due to the reported significant antimicrobial activity brought about by transition metal ions [1, 4]. The synthesis procedure has been summarized in Scheme 1. It was predicted that the stoichiometric ratio of the ligand to the metal salt would be 2 : 1 with the proposed structure of the complex shown in Figure 1.

## 2. Materials and Methods

### 2.1. Reagents

100% hydrazine hydrate, carbon disulphide, potassium hydroxide, and 4-chloroacetophenone were obtained from Merck (Germany), while 80% hydrazine hydrate, cadmium acetate, and copper acetate were obtained from R&M Marketing (UK). Benzyl chloride and 2-chloroacetophenone were obtained from Acros (Belgium) while absolute ethanol, zinc acetate, and copper acetate were obtained from Friedemann schmidt.

### 2.2. Physical Measurements

The IR spectra were recorded by a Perkin Elmer FTIR spectrophotometer within the range (4000–400 cm^−1^) using KBr discs while the melting point has been measured using an Electrothermal IA9100 digital m.p. apparatus measuring within a range of (0°C–400°C).

### 2.3. Preparation of SBDTC

#### 2.3.1. Using 100% Hydrazine Hydrate

The synthesis of SBDTC was carried out as previously reported [5]. 11.4 g (0.20 moles) of KOH was dissolved in 70 mL of 90% absolute ethanol. 10 g (0.31 moles) of 100% N_2_H_4_ was added to the solution and the mixture cooled to 0°C in an ice-salt bath. 15.2 g (0.20 moles) of CS_2_ was added dropwise via a burette to the mixture while the mixture was still being kept in the ice bath, and vigorous mixing was performed via a mechanical stirrer. After constant stirring for 1 hour, 2 layers were formed which were separated using a separating funnel, and the lower layer was later dissolved in 60 mL of cold 40% absolute ethanol maintained at 5–7°C. The mixture was then kept in an ice-bath again and 25 g (0.20 moles) of PhCH_2_Cl was added dropwise via a burette with vigorous stirring. After the PhCH_2_Cl has been completely added, the stirring was continued for 30 minutes more. The milky mixture formed was then filtered by suction filtration and washed with H_2_O and finally left to dry over Silica gel.

#### 2.3.2. Using 80% Hydrazine Hydrate

The method used was similar to SBDTC synthesis using 100% N_2_H_4_; however, some modifications needed to be implemented where 13 g (0.41 moles) of 80% N_2_H_4_ was used and the time needed for stirring after the complete addition of PhCH_2_Cl was 2 hours.

### 2.4. Synthesis of the Schiff Base Ligands

The method used for synthesis was a modified form of the one reported in [1] and the general synthesis procedure for the ligands has been summarized in Scheme 1.

#### 2.4.1. NS4 Ligand

1.98 g (0.01 moles) of SBDTC was dissolved in 40 mL of absolute ethanol and then heated on a heating plate with constant stirring in order to ensure the complete dissolving of the SBDTC. Similarly, 1.30 mL (0.01 moles) of 4-chloroacetophenone was mixed with 40 mL of absolute ethanol and heated on a heating plate for 10 minutes, which was later followed by mixing both of the reactants followed by the addition of 2–4 drops of concentrated H_2_SO_4_. The mixture was kept on the heating plate for 5 more minutes and then cooled to 0°C in an ice bath until the Schiff base precipitated. The Schiff base precipitated was filtered via suction filtration and washed with cold ethanol and dried over silica gel.

#### 2.4.2. NS2 Ligand

Method used was similar to the one used for NS4 synthesis; however, 2-chloroacetophenone was mixed with 20 mL of absolute ethanol and at the final stage, crystals were produced by slow heating to evaporate.

### 2.5. General Method for Synthesis of the Metal Complexes

The method used for synthesis was similar to the one reported in [1]. The Schiff base was dissolved in 50 mL of absolute ethanol followed by the addition of an equimolar amount of KOH and mixture was heated over a heating plate and stirred until the compounds have been completely dissolved. The solution was then treated with a stoichiometric amount of metal salt dissolved in 50 mL of absolute ethanol followed by heating using a heating plate for 5 minutes and then kept in an ice-salt bath. Finally, the product was isolated via suction filtration and washed with ethanol and dried over silica gel.

### 2.6. X-Ray Structure Determination of NS4

The crystals for analysis were prepared by the slow evaporation method where pale yellow crystals were formed. One of these crystals was selected later to be mounted on a SMART CCD diffractometer with reflection data measured at 20°C and the source of X-ray was graphite monochromated copper radiation that produced X-ray with a characteristic wavelength of 1.54180 Å. The detector was at a distance of 4 cm and a swing angle of −35°. The collected data were reduced using the program SAINT [6] and an empirical absorption correction was carried out using SADABS [7]. The structure was solved by direct methods and refined by using the full-matrix least-squares method on *F*_obs_^2^ using the SHELXTL [8] software package. All non-H atoms were anisotropically refined. The hydrogen atoms were located by difference syntheses and refined isotropically. The molecular graphics were created using mercury [9]. Atomic scattering factors and anomalous dispersion correction were taken from the International Table for X-ray Crystallography. Crystallization of NS4 from ethanol gave pale yellow crystals after 10 days via the slow evaporation method and details of the X-ray crystallographic analysis have been summarized in Table 1.

### 2.7. Antimicrobial Assay

#### 2.7.1. Target Microbes

*Staphylococcus aureus* (*S. aureus*) (11632), *Kocuria rhizophila* (*K. rhizophila*) (9341), *Bacillus cereus* (*B. cereus*) (10876), *Citrobacter freundii* (*C. freundii*) (8090), *Pseudomonas aeruginosa* (*P. aeruginosa*) (10145), *Escherichia coli* (*E. coli*) (8739), and *Aspergillus niger* (16888).

#### 2.7.2. Qualitative Antimicrobial Assay

The method that has been used for the antibacterial assay was the disc diffusion assay reported in [10] with some modifications, while the antifungal assay was also performed in a way similar to the disc diffusion assay with slight modifications. The positive controls used against bacteria were SBDTC, streptomycin, neomycin, and chloramphenicol, while amphotericin B and SBDTC were the positive controls used against the fungus. Dimethyl sulfoxide (DMSO) was used as a negative control against all the microbes investigated.

#### 2.7.3. Quantitative Antimicrobial Assay

The quantitative antimicrobial assay has been carried out by determining the minimum inhibitory concentration (MIC, mg/mL) of the compounds which is defined as the lowest concentration that managed to inhibit visible microbial growth. The method carried out to measure the MIC was quite similar to the disc diffusion assay method used in the qualitative antimicrobial assay. However, the assay was not performed on all compounds as only those compounds that caused inhibition zones of diameters greater than or equal to 9 mm (i.e., moderate effect) were considered and only bacteria were considered for this assay. 

The concentrations were prepared by two fold serial dilutions of the synthesized compounds dissolved in DMSO starting from 100 mg/mL to 6.25 mg/mL.

## 3. Results and Discussion

### 3.1. Physical Properties of the Schiff Base Ligands and Complexes

See Table 2.

### 3.2. IR Data Analysis

See Table 3.

#### 3.2.1. Schiff Base Ligands

**Figure 2:**
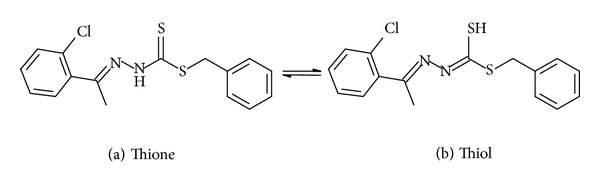
Thione-thiol tautomerism.

The peaks at 3436 cm^−1^ and 3447 cm^−1^ found in NS4 and NS2 spectra, respectively, are attributed to the presence of a secondary amine group; however, unlike SBDTC, the peaks found at around 3166 cm^−1^ and 3285 cm^−1^ in NS4 and NS2 spectra cannot be assigned to the primary amine group. Instead, it can refer to the aromatic (C–H), and this shows that the primary amine no longer exists and the reaction of SBDTC with each of the ketones took place. The peaks at 1590 cm^−1^ and 1638 cm^−1^ found in the spectra of NS2 and NS4, respectively, can be assigned to (C=N) which confirms the formation of the Schiff base ligands. The peaks at around 1023 cm^−1^ and 1051 cm^−1^ can be assigned to (N–N) of NS2 and NS4, respectively. The fact that the Schiff base ligands have been derived from SBDTC might cause them to exhibit thione-thiol tautomerism as shown in Figure 2. This happens due to the protons that are adjacent to the thione groups, as these groups are relatively unstable in the monomeric forms and tend to go to the more stable thiol form via enethiolization [4], and depending on the IR spectral information, it might be possible to confirm in which form the ligands are existing. Therefore, it can be assumed that the Schiff base ligands existed in their thione form due to the existence of a peak for (N–H) and (C=S).

#### 3.2.2. Schiff Base Metal Complexes

The broad peaks at around 3400 cm^−1^ to 3450 cm^−1^ that were found in all of the spectra for the metal complexes are attributed to water coordination with the metal ion as the peak is very broad and cannot be attributed to the secondary amine. The peaks between 1400 cm^−1^ and 1580 cm^−1^ found in all the spectra of the metal complexes can be assigned to the imine (C=N) group and the negative shift in the absorption frequency of the imine (C=N) group in the complexes relative to their Schiff base ligands proves that the imine group participates in the complexation process. Moreover, most of the IR spectra of the complexes show peaks between 1000 cm^−1^ and 1120 cm^−1^ which can be assigned to the (N–N) group and the slight increase in the absorption frequency of the (N–N) group might be due to the decrease in repulsion between the two nitrogen atoms. This is due to the fact that the nitrogen atom of the imine group forms a coordinate bond with the metal ion; therefore, the pair of electrons used to form the bond are no longer in close proximity with the electrons of the adjacent nitrogen atom leading to a decrease in repulsion [11].

There is an absence of a peak with reasonable intensity at 950–970 cm^−1^ which indicates the absence of the (C=S) group and this proves that the Schiff base ligand forms a complex with the metal ion through its thiolate group and not through its thioketo sulfur. This would also mean that in solution, the ligand exists in its thiol form and this is proven further by the absence of the (N–H) group.

**Figure 3:**
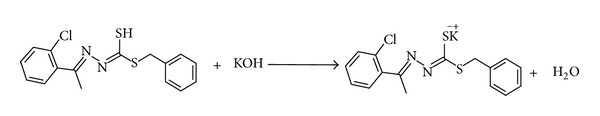
Deprotonation of the thiol by KOH.

In the presence of KOH, the thiol proton undergoes deprotonation forming thiolate anions (Figure 3) in solution enhancing the nucleophilicity of the ligand and affording a uninegative chelation with metal ions [5].

In an attempt to synthesize the metal complex without the presence of KOH, two crucial points were deduced from this unsuccessful attempt. This attempt confirmed the fact that the ligand was not in the thione form in solution as if it did, there would be no need for deprotonation by KOH and the attempt would have been successful. Secondly, it confirms the initial assumption that the ligand chelates with metal ion via an ionic bond between the negatively charged deprotonated thiol group and the metal ion. On the contrary, when KOH was added, the attempt was successful due to the deprotonation of the thiol group. Therefore, it can be deduced that the metal complex structure would be a bischelated bidentate complex as shown in Figure 1.

### 3.3. X-Ray Crystallography Analysis of NS4

**Figure 4:**
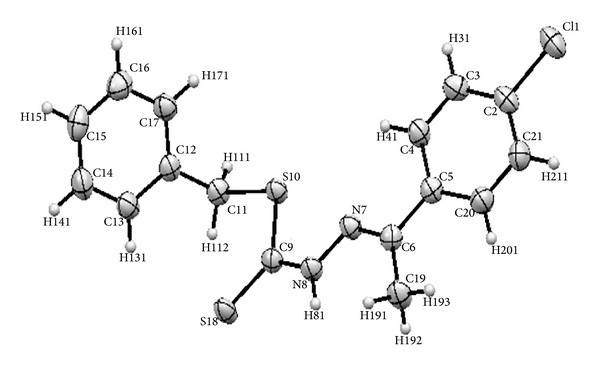
ORTEP plot of NS4 showing 50% probability displacement ellipsoids in addition to the atomic numbering scheme.

Figure 4 shows the structure and atomic numbering of the Schiff base ligand while Tables 4 and 5 give the most vital bond lengths and angles of the crystal structure.

The characteristic bond length is that of Cl1–C2 possessing a bond length of 1.75 Å. This is the first chlorine-carbon bond length to be reported in a dithiocarbazate derived compound at the time the experiment was carried out. The bond N8–C9 had a length of 1.34 Å while C6–N7 had a length of 1.29 Å which is shorter than that of N8–C9 due to the double bond character of the latter indicating that it is an imine functional group. The case is similar when comparing the bonds C9–S10 and C9–S18 with bond lengths of 1.77 Å and 1.66 Å, respectively, with the latter having a shorter bond length confirming its double bond character, thus indicating that the ligand existed in its thione form. The bond lengths of imine group C6–N7 and that of C9–S18 are similar to the ones found in the previous literature [1–3], which indicates that these bond lengths are typical of Schiff base compounds derived from dithiocarbazates. However, the N7–N8 bond length of 1.37 Å is shorter than that of the unsubstituted SBDTC which possesses an N–N bond length of 1.406 Å [1] and that could be due to the delocalization of electrons from the benzene ring of the chloroacetophenone moiety. 

**Figure 5:**
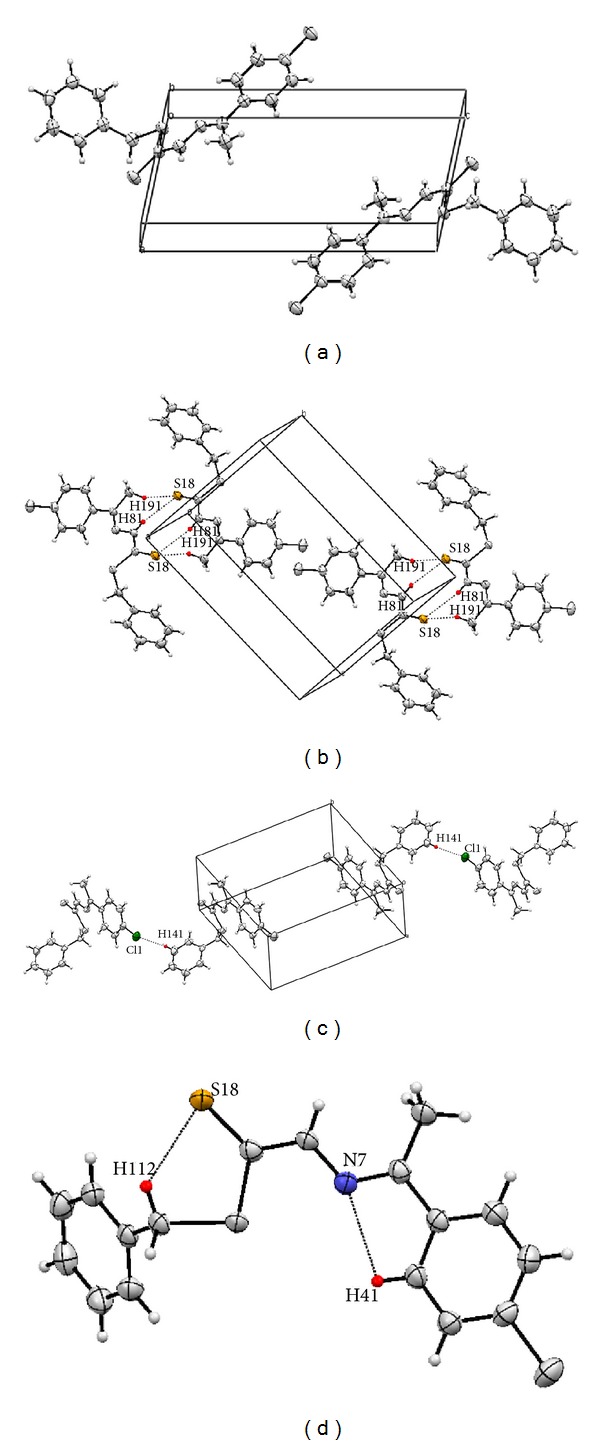
(a) Packing of molecules per unit cell viewed along the *b*-axis. (b) Packing diagram of NS4 showing the H81–S18 and H191–S18 hydrogen bond interactions. (c) Packing diagram of NS4 showing the Cl1–H141 hydrogen bond interactions. (d) Intramolecular hydrogen bond interactions within NS4 molecules.

The structure geometry is considered to be planar except for the benzene ring derived from SBDTC which is out of plane and is nearly perpendicular to the plane with a torsion angle of 86.8°. The molecules seem to be stabilized by intramolecular and intermolecular H81–S18, H191–S18, and Cl1–H141 hydrogen bond interactions (Table 6) (Figure 5).

### 3.4. Antimicrobial Assay

#### 3.4.1. Qualitative Antimicrobial Assay

Table 7(a) shows a summary of the mean results obtained for the inhibition zones caused by the Schiff base complexes and ligands, while Table 7(b) shows the mean results for the inhibition zones caused by reference compounds and controls.

NS2 has inhibited the growth of all types of bacteria strongly with the strongest inhibition observed against *B. cereus* with an inhibition zone of 21.63 mm; however, the compound showed no inhibition against the fungus *Aspergillus niger*. Generally, Zn(NS2)_2_ has shown a strong effect on bacteria especially *K. rhizophila*; however, it has shown its highest level of inhibition against the fungus *Aspergillus niger* with inhibition zone of 23.15 mm. Cd(NS2)_2_ had weak effect on both Gram-positive and Gram-negative bacteria except *K. rhizophila* and *P. aeruginosa*; however, the greatest effect has been observed with *Aspergillus niger* with an inhibition zone of 29.98 mm. Moreover, Ni(NS2)_2_ showed weak activity on Gram-negative bacteria with no effect observed on *E. coli* and *C. freundii*. The case is quite similar for Gram-positive bacteria where the only exception was a moderate effect on *K. rhizophila*. However, the largest inhibition zone has been found with the fungus *Aspergillus niger* with an inhibition zone of 17.17 mm indicating its selective antifungal activity. Cu(NS2)_2_ showed no effect on any of the bacterial strains tested except for an insignificant effect of inhibition zone less than 8 mm on *S. aureus* and *P. aeruginosa*; moreover, this complex had no effect against the fungus *Aspergillus niger*.

NS4 seems to possess a very low antimicrobial activity, and the case was similar to Zn(NS4)_2_. Cd(NS4)_2_ showed lack of antifungal activity and a very low and insignificant effect on Gram-negative bacteria and a slightly higher effect has been seen with Gram-positive bacteria although these effects were insignificant. However, there was a very high effect exerted on *K. rhizophila* with an inhibition zone of 30.05 mm. Ni(NS4)_2_ has acted weakly on Gram-negative bacteria with moderate effect on *C. freundii*, but no antifungal activity. However, Ni(NS4)_2_ generally acted better on Gram-positive bacteria with moderate effects on all of those under investigation. Cu(NS4)_2_ possessed weak antimicrobial activity and also lacked antifungal activity.

It can be deduced from the results that the activity of the NS4 complexes is generally higher than that of the Schiff base ligand which might be explained via Overtone's concept and chelation theory [12]. The Overtone's concept of cell permeability states that the cell membrane surrounding the microbial cell favours the passage of lipid soluble particles causing lipid solubility to be a crucial factor in determining antimicrobial activity and chelation tends to reduce the polarity of the metal ion due to the overlap of the ligand orbital and partial sharing of positive charge with the donor groups. Furthermore, it increases the delocalization of *π*-electrons over the whole chelation ring, increasing the lipophilicity of the complex and consequently enhancing its penetration to the cell. Moreover, it is also suspected that other factors might influence the activity of the compounds such as solubility, conductivity, dipole moment, and cell permeability mechanisms that are influenced by the presence of metal ions. However, NS2 did not follow this concept as it possessed a higher antibacterial activity than that of its complexes and this phenomenon can only be explained via the assumption mentioned previously regarding the effect of solubility on the antimicrobial activity of the compounds. The NS2 Schiff base ligand was very soluble in the solvent DMSO; however, its metal complexes were partially soluble resulting in compound concentrations less than the intended ones which consequently affected their antimicrobial activities.

The results show that Gram-positive bacteria were inhibited more strongly than Gram-negative bacteria and that can be explained by considering the structural features of both bacterial types. Gram-negative bacteria possess an extra outer layer on top of the peptidoglycan and this has been found to be highly impermeable. Moreover, Gram-positive bacteria have polysaccharides in their cell wall called teichoic acids, which are negatively charged and have facilitated the passage of the positive metal ions. The position of chlorine in the benzene ring is crucial as it affected the activity of the ligand where it showed that NS2 has had a much higher activity than that of NS4. This might suggest that the binding pocket of the target in the bacteria favours the orthoposition of the chlorine. Despite the fact that NS2 and NS4 derivatives acted with variable strengths on the microbes tested, it has been found that the strongest inhibition level observed was by the Cd complexes of both ligands against *K. rhizophila*. This suggests that there is a region in the binding pocket of *K. rhizophila* that interacts specifically with Cd^2+^ ion and it also suggests that the interaction is not affected strongly by the position of the Cl atom on the benzene ring, as the inhibition zones caused by both of the Cd complexes from both ligands were similar.

#### 3.4.2. Comparison between the Antimicrobial Activity of Synthesized and Reference Compounds

The activities of the synthesized compounds were compared with those of the reference compounds, by forming a ratio of the mean inhibition diameter of the synthesized compound to that of the reference compound (Table 8).

It can be deduced from (Table 8) that NS2 acted nearly with the same strength as the positive controls except in chloramphenicol and neomycin against *S. aureus* and *P. aeruginosa,* respectively. However, it proved to be 1.5 times stronger than streptomycin and SBDTC in their activity against *K. rhizophila*. The compound had no antifungal activity.

Zn(NS2)_2_ showed generally similar strength to the positive controls against all bacteria and the fungus except *E. coli*; however, it showed 1.5 times more strength than streptomycin against *K. rhizophila*. Cd(NS2)_2_ exerted half the strength of the positive controls against *S. aureus*, *B. cereus*, *E. coli,* and *C. freundii*; however, it acted with similar strength or more against the remaining bacterial types. Antifungal activity was higher than that of the control amphotericin B. Ni(NS2)_2_ showed the weakest effects relative to the positive controls, where, unlike the positive controls, no effect has been observed against *C. freundii* and *E. coli*. Similar activities to, or half, the positive controls have been observed with the remaining bacteria and fungus. The case was worse with Cu(NS2)_2_ which possessed no activity relative to the positive controls against most of the microbial species except an effect with half of the strength relative to the positive controls against *S. aureus* and *P. aeruginosa*. The case was also similar to NS4 which only had weaker effects than those of the controls against *B. cereus* and *C. freundii* only.

Zn(NS4)_2_, Cd(NS4)_2_, Ni(NS4)_2_, and Cu(NS4)_2_ complexes showed generally an effect that is half the strength of the positive controls or slightly higher against *S. aureus*, *B. cereus*, *K. rhizophila,* and *C. freundii*, with no effect against the other microbial species. However, the ligand had an effect on *B. cereus* and *C. freundii* specifically, with low activity relative to the antibiotics against *B. cereus* but exerted activity with half the strength of the antibiotics against *C. freundii*.

#### 3.4.3. Effect of Chloroacetophenone Analogue on Antimicrobial Activity

The effect of chloroacetophenone analogues on microbial activity can be analysed via comparing the results obtained from this experiment with the previous literature where a different ketone has been reacted with SBDTC. In order to attribute any differences in results to the structure that has been derived from the ketone. The parameter used for the comparison is the inhibition zone diameter.

**Figure 6:**
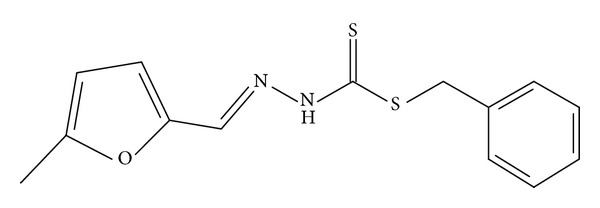


The compound synthesized by Tarafder et al. [11] in Figure 6 was found to lack activity against *P. aeruginosa* and a similar result has been found in NS4; however, NS2 exerted a moderate effect with an inhibition zone diameter of 13.88 mm indicating that 2-chloroacetophenone analogue has enhanced antibacterial activity.

**Figure 7:**
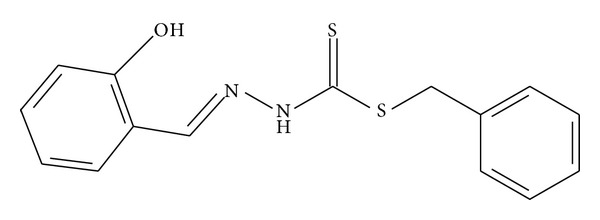


The compound synthesized by Hossain et al. [4] in Figure 7 caused an inhibition zone diameter of 10 mm when tested against *P. aeruginosa* which is still less than the diameter of 13.88 mm caused by NS2, showing that 2-chloroacetophenone analogue enhances the activity of the compound. The case was different with NS4 which showed no activity against *P. aeruginosa* suggesting the negative effect of the 4-chloroacetophenone analogue. Moreover, a similar trend was observed with *B. cereus* where NS2 caused a larger inhibition zone than the compound in Figure 7 with diameters of 21.63 mm and 12 mm, respectively, but NS4 caused a smaller diameter of 6.53 mm.

**Figure 8:**
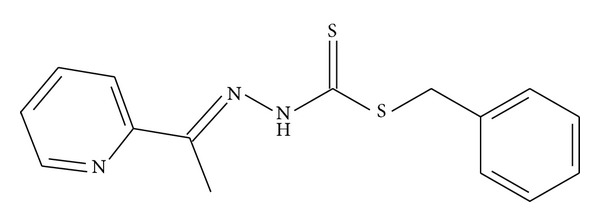


The compound synthesized by Tarafder et al. [13] in Figure 8 exerted a zone inhibition of 7 mm against *P. aeruginosa* which is less than the diameter caused by NS2 suggesting that the 2-chloroacetophenone analogue enhanced antimicrobial activity. 

### 3.5. Quantitative Antimicrobial Assay

The activity of the bacteria has been assessed quantitatively via measuring the minimum inhibitory concentration (MIC). Some of the MIC of the controls used was obtained from the previous literature [14–23]. MIC values were summarized in (Table 9).

Ni(NS4)_2_ was the weakest compound relative to the antibiotics with MIC values equal to or greater than 25 mg/mL. NS2 and Zn(NS2)_2_ had relatively the lowest MIC values of <6.25 mg/mL for *B. cereus* and were thus considered the most active. Generally, the compounds examined against *B. cereus* were much weaker than the commercial antibiotics; however NS2 and Zn(NS2)_2_ might be comparable to neomycin which had a value of 1.10 mg/mL. Cd(NS4)_2_ was the most active against *K. rhizophila* while *E. coli* has been inhibited strongly by NS2 and Zn(NS2)_2_ with a value of <6.25 mg/mL._._ *P. aeruginosa* was inhibited mostly by Zn(NS2)_2_ and Cd(NS2)_2_ and it seems that NS2 was much weaker than the antibiotics used for the investigation. *C. freundii* was inhibited mostly by NS2, Zn(NS2)_2_, and Cu(NS4)_2_ with MIC values of <6.25 mg/mL; however, the compounds were generally much weaker than the antibiotics used for the investigation. The results of the quantitative analysis confirmed the fact that NS2 has stronger antimicrobial activity than that of NS4.

## 4. Conclusion

Characterization of the synthesized compounds has revealed that the complexes are bidentate having nitrogen and sulfur donor atoms, while the X-ray analysis of NS4 revealed a planar structure with an out-of-plane benzyl ring. Qualitative antimicrobial assay confirmed the potential of NS2 and its complexes as strong antimicrobial agents and the generally higher biological activity possessed by NS2 when compared to NS4. It has been noted that the Cd complexes of NS2 and NS4 acted strongly specifically on *K. rhizophila*. Moreover, the qualitative assay has shown that most of the compounds exerted effects that were similar in strength or even greater than some of the known antibiotics which reflects their strength. The quantitative assay requires further dilutions of the synthesized compounds in order to enable a more accurate comparison between their strength and that of the antibiotics, but it confirmed the stronger action of NS2 derivatives over NS4 and also the comparable action of NS2 and Zn(NS2)_2_ to that of neomycin against *B. cereus*.

## Figures and Tables

**Table 1:** Crystallographic data and structure refinement details of NS4.

Empirical formula	C_16_H_15_ClN_2_S_2_
Formula weight	334.89
Temperature (K)	150
Crystal class	Triclinic
Space group	*P*-1
Unit cell dimensions	
*a* (Å)	6.2206(7)
*b* (Å)	9.7222(10)
*c* (Å)	13.5943(14)
*α* (°)	89.729(8)
*β* (°)	101.960(9)
*γ* (°)	97.447(9)
Volume (Å^3^)	797.32(15)
*Z*	2
*ρ*_calc_ (g cm^−3^)	1.39
Radiation type	Cu K*α*
Wavelength (Å)	1.5418
Crystal size (mm)	0.06 × 0.08 × 0.18
*θ* Range for data collection (°)	3–72
Reflections measured/independent	7883/2834 (*R*_int⁡_ = 0.0327)
*θ*_max⁡_	71.6937
Limiting indices	−7 ≤ *h* ≤ 7
	−11 ≤ *k* ≤ 11
	−16 ≤ *l* ≤ 16
Refinement on	*F*
*R*-factor	0.066
*R*_*w*_-factor	0.178
Goodness of fit (S)	0.97
Minimum and maximum residual electron density (eÅ^−3^)	−0.45 and 0.80
Reflections used	2821
Number of parameters	190

**Table 2:** Physical properties and yield of the Schiff bases and their complexes.

Compound	Colour	Yield	Melting point (°C)
SBDTC	White	7.67 g	124
NS4	Light yellow	1.46 g	138.5
NS2	White	0.61 g	178
Zn(NS4)_2_	Yellowish green	0.65 g	200
Cd(NS4)_2_	Yellowish brown	2.81 g	189
Ni(NS4)_2_	Dark green	0.52 g	Decomposes at 309.5
Cu(NS4)_2_	Dark brown	0.52 g	190
Zn(NS2)_2_	White	0.35 g	159
Cd(NS2)_2_	Greyish green	0.59 g	Decomposes at 170
Ni(NS2)_2_	Light green	0.12 g	Decomposes at 160
Cu(NS2)_2_	Dark brown	0.10 g	Decomposes at 205

**Table 3:** IR spectral data of SBDTC, free ligand, and their complexes.

Compound	Infrared absorption bands (frequency, cm^−1^)							
	*ν*(NH)	*ν*(NH_2_)	*ν*(C=S)	*ν*(N–N)	*ν*(C=N)	*ν*(M–N)*	*ν*(O–H)	*ν*(Other bands)
SBDTC	3451	3250, 3172	950	1048	—	—	—	2 peaks at 710, 698

NS4	3436	—	950	1051	1638	—	—	(i) Aromatic C–H at 3166 (ii) Peak at 827 (iii) Peak at 704

NS2	3447	—	972	1023	1590	—	—	(i) Aromatic C–H at 3285 (ii) Peak at 704 (iii) Peak at 622

Zn(NS4)_2_	—	—	—	1000	1400	Obscured by other peaks	3432	Broad peak ~(500–900)

Cd(NS4)_2_	—	—	—	1086	1450	600	3430	(i) Peak at 822 (ii) Peak at 700

Zn(NS2)_2_	—	—	—	1121	1578	750	3436	(i) Peak at 697 (ii) Peak at 619

Cd(NS2)_2_	—	—	—	1120	1421	Obscured by other peaks	3448	(i) Peak at 700 (ii) Peak at 619

Ni(NS4)_2_	—	—	—	1116	1400	500	3400	(i) Peak at 827 (ii) Peak at 703

Cu(NS4)_2_	—	—	—	1117	1406	619	3420	(i) Peak at 825 (ii) Peak at 695

Cu(NS2)_2_	—	—	—	1120	1406	Obscured by other peaks	3410	Peak at 619

Ni(NS2)_2_	—	—	—	1110	1421	510	3430	Peak at 620

(M–N)*: this refers to the dative bond that is formed between the metal ion (M) and the nitrogen (N) atom.

**Table 4:** Summary of selected bond lengths found in NS4.

Bond lengths (Å)			
Cl1–C2	1.746(4)	C6–N7	1.285(5)
C2–C21	1.381(6)	N7–N8	1.370(4)
C3–H31	0.938	N8–H81	0.870
C5–C20	1.387(6)	C9–S18	1.660(4)
C9–S10	1.765(4)	N8–C9	1.343(5)
S10–C11	1.825(4)		

**Table 5:** Summary of the bond angles in NS4.

Bond angles (°)			
Cl1–C2–C3	119.0(3)°	C6–N7–N8	119.1(3)°
N7–C6–C19	124.1(3)°	N7–N8–H81	120.673°
N7–N8–C9	118.7(3)°	N8–C9–S10	112.9(3)°
N8–C9–S18	122.3(3)°	S10–C9–S18	124.8(2)°
C9–S10–C11	102.76(17)°	S10–C11–C12	115.4(3)°

**Table 6:** Hydrogen bond geometry (Å, °).

D–H⋯A	D–H	H⋯A	D⋯A	D–H⋯A
C14–H141⋯Cl1^i^	0.94	2.88	3.813(5)	173
N8–H81⋯S18^ii^	0.87	2.67	3.526(3)	166
C19–H191⋯S18^ii^	0.98	2.80	3.440(4)	124
C11–H112⋯S18 (intramolecular)	0.98	2.65	3.145(4)	112
C4–H41⋯N7 (intramolecular)	0.94	2.41	2.742(5)	100

Symmetry codes: ^i^ *x* + 1, *y*, *z* − 1; ^ii^−*x* + 1, −*y*, −*z*.

**Table:** (a)

Compound	Gram-positive bacteria			Gram-negative bacteria			Fungus
	*S. aureus *	*B. cereus *	*K. rhizophila *	*E. coli *	*P. aeruginosa *	*C. freundii *	*Aspergillus niger *
NS2	17.60	21.63	19.01	15.62	13.88	19.60	—
Zn(NS2)_2_	16.23	18.12	18.34	8.60	13.63	16.99	23.15
Cd(NS2)_2_	8.90	8.16	25.76	7.43	15.27	8.33	29.98
Ni(NS2)_2_	7.40	8.51	13.53	—	8.88	—	17.17
Cu(NS2)_2_	7.09	—	—	—	7.45	—	—
NS4	—	6.53	—	—	—	11.50	—
Zn(NS4)_2_	9.19	8.17	7.40	—	—	9.69	—
Cd(NS4)_2_	7.77	6.61	30.05	—	—	6.81	—
Ni(NS4)_2_	10.87	10.22	9.13	—	—	12.99	—
Cu(NS4)_2_	10.33	6.64	8.21	—	—	9.99	—

**Table:** (b)

Controls and reference compounds	Gram-positive bacteria			Gram-negative bacteria			Fungus
	*S. aureus *	*B. cereus *	*K. rhizophila *	*E. coli *	*P. aeruginosa *	*C. freundii *	*Aspergillus niger *
DMSO	—	—	—	—	—	—	—
Streptomycin	14.08	15.67	12.20	14.44	13.54	Not available	
Neomycin	18.14	18.52	18.57	16.58	21.87	18.91	
Chloramphenicol	25.86	21.70	25.87	—	15.01	19.80	
SBDTC	19.07	22.73	13.33	15.70	14.90	21.19	18.57
Amphotericin B							19.17

*Zones of inhibition of diameters 14 mm and above were considered to be significant, 9 mm–14 mm were considered moderate, and less than 9 mm were considered weak and insignificant. (—) refers to absence of measurable inhibitory action.

**Table 8:** Ratio of mean inhibition zone diameter of synthesized compounds to that of reference compounds.

	*S. aureus *	*B. cereus *	*K. rhizophila *	*E. coli *	*P. aeruginosa *	*C. freundii *	*Aspergillus niger *
NS2							
Streptomycin	1.25	1.38	1.56	1.08	1.03	Not available	
Neomycin	0.97	1.17	1.02	0.94	0.63	1.04	
Chloramphenicol	0.68	1.00	0.73	Control has no effect	0.92	0.99	
SBDTC	0.92	0.95	1.43	0.99	0.93	0.92	0.00
Amphotericin B							0.00
Zn(NS2)_2_							
Streptomycin	1.15	1.16	1.50	0.60	1.00	Not available	
Neomycin	0.89	0.98	0.99	0.52	0.62	0.90	
Chloramphenicol	0.63	0.84	0.71	Control has no effect	0.91	0.86	
SBDTC	0.85	0.80	1.38	0.55	0.91	0.80	1.25
Amphotericin B							1.21
Cd(NS2)_2_							
Streptomycin	0.63	0.52	2.11	0.51	1.13	Not available	
Neomycin	0.49	0.44	1.39	0.45	0.70	0.44	
Chloramphenicol	0.34	0.38	1.00	Control has no effect	1.02	0.42	
SBDTC	0.47	0.36	1.93	0.47	1.02	0.39	1.61
Amphotericin B							1.56
Ni(NS2)_2_							
Streptomycin	0.53	0.54	1.11	0.00	0.66	Not available	
Neomycin	0.41	0.46	0.73	0.00	0.41	0.00	
Chloramphenicol	0.29	0.39	0.52	Control has no effect	0.59	0.00	
SBDTC	0.39	0.37	1.02	0.00	0.60	0.00	0.92
Amphotericin B							0.90
Cu(NS2)_2_							
Streptomycin	0.50	0.00	0.00	0.00	0.55	Not available	
Neomycin	0.39	0.00	0.00	0.00	0.34	0.00	
Chloramphenicol	0.27	0.00	0.00	Control has no effect	0.50	0.00	
SBDTC	0.37	0.00	0.00	0.00	0.50	0.00	0.00
Amphotericin B							0.00
NS4							
Streptomycin	0.00	0.42	0.00	0.00	0.00	Not available	
Neomycin	0.00	0.35	0.00	0.00	0.00	0.61	
Chloramphenicol	0.00	0.30	0.00	Control has no effect	0.00	0.58	
SBDTC	0.00	0.29	0.00	0.00	0.00	0.54	0.00
Amphotericin B							0.00
Zn(NS4)_2_							
Streptomycin	0.65	0.52	0.61	0.00	0.00	Not available	
Neomycin	0.51	0.44	0.40	0.00	0.00	0.51	
Chloramphenicol	0.36	0.38	0.29	Control has no effect	0.00	0.49	
SBDTC	0.48	0.36	0.56	0.00	0.00	0.46	0.00
Amphotericin B							0.00
Cd(NS4)_2_							
Streptomycin	0.55	0.42	2.46	0.00	0.00	Not available	
Neomycin	0.43	0.36	1.62	0.00	0.00	0.36	
Chloramphenicol	0.30	0.30	1.16	Control has no effect	0.00	0.34	
SBDTC	0.41	0.29	2.25	0.00	0.00	0.32	0.00
Amphotericin B							0.00
Ni(NS4)_2_							
Streptomycin	0.77	0.65	0.75	0.00	0.00	Not available	
Neomycin	0.60	0.55	0.49	0.00	0.00	0.69	
Chloramphenicol	0.42	0.47	0.35	Control has no effect	0.00	0.66	
SBDTC	0.57	0.45	0.68	0.00	0.00	0.61	0.00
Amphotericin B							0.00
Cu(NS4)_2_							
Streptomycin	0.73	0.42	0.67	0.00	0.00	Not available	
Neomycin	0.57	0.36	0.44	0.00	0.00	0.53	
Chloramphenicol	0.40	0.31	0.32	Control has no effect	0.00	0.50	
SBDTC	0.54	0.29	0.62	0.00	0.00	0.47	0.00
Amphotericin B							0.00

**Table 9:** Shows the minimum inhibitory concentration (MIC) of the compounds.

Compound	MIC (mg/mL)					
	*S. aureus *	*B. cereus *	*K. rhizophila *	*E. coli *	*P. aeruginosa *	*C. freundii *
NS2	<6.25	<6.25	25	<6.25	50	<6.25
Zn(NS2)_2_	<6.25	<6.25	12.50	<6.25	<6.25	<6.25
Cd(NS2)_2_	<6.25	—	100	—	<6.25	—
Ni(NS2)_2_	—	100	100	—	—	—
Cu(NS2)_2_	—	—	—	—	—	—
NS4	—	—	—	—	—	12.50
Zn(NS4)_2_	—	—	—	—	—	50
Cd(NS4)_2_	—	—	<6.25	—	—	—
Ni(NS4)_2_	25	—	—	—	—	50
Cu(NS4)_2_	<6.25	—	—	—	—	<6.25
SBDTC	<6.25	<6.25	<6.25	<6.25	0.78*	<6.25
Streptomycin	0.001563*	0.000391*	>0.10*	0.0008*	0.004*	0.004*
Neomycin	N/A	1.10*	0.0039*	0.0064*	N/A	0.032*
Chloramphenicol	0.005*	0.01*	0.05*	0.004*	0.02*	0.002*

— refers to the absence of an MIC value for a certain compound as it was not investigated for its MIC due to its low activity. *refers to an MIC value that was obtained from the previous literature or experiments. N/A: (not available).
